# The relationships between language, functional hearing, social, and motor development skills in children with early cochlear implants

**DOI:** 10.1007/s00405-024-08635-8

**Published:** 2024-04-17

**Authors:** Şevval Utku Arat, Merve İkiz Bozsoy, Esra Yücel

**Affiliations:** Department of Audiology, Faculty of Health Sciences, Hacettepe University, Ankara, 06100 Turkey

**Keywords:** Cochlear implant, Language, Motor development, Social skills, Functional hearing

## Abstract

**Purpose:**

The purpose of this study was to investigate the relations between functional hearing, language, social, bilateral coordination and manual dexterity skills in children with early cochlear implants (CIs).

**Methods:**

Thirty children with CIs were included in this study. The manual dexterity and bilateral coordination development of the participants were evaluated with Manual Dexterity and Bilateral Coordination subtests of Bruininks-Oseretsky Motor Proficiency-2 (BOT-2). Their language skills were assessed by the Test of Early language Development-3. To assess the functional hearing of participants the Functioning After Pediatric Cochlear Implantation scale (FAPCI) was administered their caregivers. Also, the Social Skills Evaluation Scale was administered to participants’ teachers to asses their social skills.

**Results:**

There were significant correlations between participants’ receptive and expressive language skills, Manual Dexterity, and FAPCI scores (*p* < 0.05). There were also significant relationships between the SSES and FAPCI scores of the participants (*p* < 0.05). However, the Bilateral Coordination subtest did not show any significant correlation with any of the measurements (*p* > 0.05).

**Conclusion:**

The results suggest that the language, manual dexterity and functional hearing abilities of children with CIs are closely related. Although, there were no significant correlations between all of the measurement, it is important to look beyond hearing and speech evaluations to assess the whole child.

## Introduction

Hearing is one of the earliest senses that develop, with the cochlea maturing by the 23rd week of gestation. Toddlers and children learn to communicate using oral language and develop cognitive skills through listening, but they can also interpret other people’s behavior and intentions [1].

Most children who are born deaf or become deaf before the age of 3 years lag behind their normal-hearing (NH) peers in spoken language skills. However, it is now known that early deafness affects nearly all aspects of general development, including cognitive, motor, and social domains [1]. The researchs suggest that prelingually deaf or hard of hearing children are at risk and may experience difficulty in developing social and emotional skills such as being able to recognize and manage emotions, appreciate the perspective of others, establish and maintain positive relationships [2, 3]. In addition, deaf children were found to be more likely to have deficiencies in motor abilities, including balance, coordination, visual-motor, and speed of movements [4, 5].

In order to prevent the negative effects of deafness, cochlear implants (CIs) have become widely accepted devices in the rehabilitation of deaf children [1]. According to research, age at implantation is an important factor in the outcomes of prelingually deaf children [6]. The rationale behind early implantation is based on neural plasticity and sensitive learning periods [7]. In human development, the first 3–4 years of life are defined as the most rapid synaptogenesis and highest central auditory nerve system plasticity. As a result, external auditory stimuli are crucial throughout the first 3–4 years of life for developing lower- and higher level neural connections [8].

It is well-known that no part of the brain works in isolation, early auditory deprivation alters the brain’s effective connectivity not only within the auditory system but also between auditory system and higher level cortex areas, such as the basal ganglia, cerebellum, entorhinal cortex, hippocampus, amygdala and frontal areas [9, 10]. These areas are crucial for motor development skills, executive functions and learning [9–11]. Recent studies revealed that bilateral coordination and manual dexterity skills involve a series of cerebral process including sensory input, perceptual and cognitive processing [12, 13]. Moreover, CI users have difficulty in cognitive skills [14]. Numerous studies have shown that early implantation improves spoken language, functional hearing and social abilities [7, 15, 16]. However, there have been few studies that investigate the relation between these abilities and bilateral coordination and manual dexterity motor developmental areas. In this study, we aimed to investigate the relations between functional hearing, language, social, bilateral coordination and manual dexterity development skills in children with early CIs.

## Material and methods

This study was conducted at Hacettepe University Faculty of Health Sciences, Audiology Department and received ethical approval from the Hacettepe University Non-Interventional Clinic Research Ethics Board (GO20/762). The participants and their parents provided informed consent on the day of enrollment.

### Participants

The study included a sample of 30 children with CIs (14 F, 16 M), aged between 4 and 6 years 11 months. The following inclusion criteria were used to determine the participants: (1) failing in the newborn hearing screening, (2) having prelingual severe to profound bilateral sensorineural hearing loss, (3) being a bilateral or unilateral CI user, (4) having the first CI surgery for up to 30 months, (5) having regular follow-up of medical, audiological, and auditory perception rehabilitation after surgery. Apart from these, children diagnosed with inner ear malformations, auditory neuropathy spectrum disorders, neurological or developmental disorders, learning difficulties, and other comorbidities were excluded.

### Test battery

#### Test of Early Language Development (TELD-3)

The Turkish version of the Test of Early Language Development (TELD-3:T) was used to evaluate participants’ receptive and expressive language development. TELD-3 was developed by Hresko, Reid and Hammill [17]. The normative data for the Turkish validity and reliability of TELD-3 consisted of 1200 normally developing children aged between 18 months and 8 years. The results showed that the validity and reliability of TELD-3:T were strong and significant. Also, TELD-3:T accurately examined receptive and expressive language, and identified children with language delay and disorders [18]. The test comprises of verbal instructions provided to the child, as well as stimuli such as objects or drawings to which the child is expected to respond. In this study, we presented TELD-3:T scores as standard scores. Receptive and expressive language scores are calculated separately. The degree of spoken language development (very good, good, above average, average, below average, poor, very poor) was determined based on the combined scores of receptive and expressive language.

#### The Functioning After Pediatric Cochlear Implantation (FAPCI)

The Functioning After Pediatric Cochlear Implantation (FAPCI) was used to evaluate the functional hearing of participants. It is a family-centered communicative performance scale based on the World Health Organization’s conceptual model of functioning. It was developed by Lin et al. [19] to provide an objective assessment of the auditory performance of children with CIs. This scale evaluates verbal communication through educational and behavioral contexts rather than language skill. It consists of 23 questions, the lowest and highest possible scores are 23 and 115, respectively.

Turkish validity and reliability study of the FAPCI was conducted by Yücel et al. [20]. It was administered to families of children aged 2 to 6 years with NH and CIs, respectively. When the total scores of children with NH and CIs were evaluated for the construct validity of the scale, a significant difference was found between the scores of the 2 groups. FAPCI internal reliability consistency was also confirmed (Cronbach’s alpha coefficient > 0.90). The statistical analysis showed that Turkish version of FAPCI is a valid and reliable tool to evaluate the communication skills of children with CIs.

#### The Social Skills Evaluation Scale (SSES)

The Social Skills Assessment Scale (SSES), developed by Avcioglu [21] in 2007, was used to assess participant's social behavior skills. In the validity and reliability study, it was administered to teachers of children aged 4 to 6 years. For the content validity, an expert’s opinion was consulted. To determine the reliability of the scale Cronbach Alpha internal consistency was calculated and it was found 0.98. In this Likert-type scale consisting of 62 questions, evaluations were performed using the responses ‘always’, ‘very often’, ‘usually’, ‘very rarely’ and ‘never’. The minimum score obtainable on the scale is 62, and the maximum score is 310. A high score on the scale indicates that the child has adequate social abilities, whereas a low score suggests a lack of sufficient social skills. In this study, teachers providing special education and rehabilitation services rated the social skills of children who had not yet started kindergarten or school, while teachers rated the social skills of children who were enrolled in kindergarten or school.

#### Bruininks–Oseretsky Test of Motor Proficiency-2 (BOT-2)

The Bruininks–Oseretsky Test of Motor Proficiency-2 (BOT-2) was used to evaluate participants’ motor development skills. This is a performance-based test that evaluates the motor development and coordination skills of individuals aged 4 to 21 [22]. The test battery consists of four main components: (1) fine motor control, (2) dexterity coordination, (3) body coordination, (4) strength and agility, with eight sub-components: (1) fine motor precision, (2) fine motor integration, (3) manual dexterity, (4) bilateral coordination, (5) balance, (6) running speed and quality, (7) upper limb coordination, and (8) strength.

The BOT-2 standardization scores are based on normative data obtained from 1520 children and adolescents aged 4 to 21 years in the United States. It has well-established validity and reliability. The BOT-2’s content validity was confirmed through test content development, and it discriminates between different clinical groups and typically developing children. Inter-rater reliability of the BOT-2 is good ranging from 0.92 to 0.99 for the composite scores and test–retest reliability is good to excellent.

Four assessment options are available for the BOT-2: (1) complete form, (2) short form, (3) selected components, (4) selected sub-components. In this study, manual dexterity (making dots in circles, transferring pennies, placing pegs into a pegboard, sorting cards, stringing blocks) and bilateral coordination (touching nose with index fingers-eye closed, jumping jacks, jumping in place-same sides synchronized, jumping in place- same sides synchronized, pivoting thumbs and index fingers, tapping feet and fingers-same sides synchronized, tapping feet and fingers-opposite sides synchronized) sub-components of the test battery were included. The raw scores of sub-components are converted to the standard scores based on age and gender using the BOT-2 manual’s normative data. The standard scores also described into five categories: well-above average, above average, average, below average, and well-below average.

### Statistical analysis

Statistical analysis was carried out using SPSS version 23. The variables’ normality was determined using histograms, probability plots, and Kolmogorov–Smirnov/Shapiro–Wilk’s test. Descriptive statistics were reported as mean, standard deviation for parametric variables, and reported as median, interquartile range (IQR) for non-parametric variables. The categorical variables were presented as frequencies and percentages. Pearson’s correlation coefficient was used to analyze the correlation between parametric variables, whereas Spearman’s correlation coefficient was used to analyze the correlation between non-parametric variables. Correlations were considered statistically significant when the *p *value was less than 0.05.

## Results

### Demographic and clinical characteristics of the participants

The sample comprised 30 children with CIs, consisting of 14 females and 16 males. The age range of the children was from 48 to 81 months, with a mean age of 60.73 ± 9.74 (months). Of the participants, 24 used bilateral CIs, while 6 used unilateral CI. Thirteen of the bilateral CIs users received their CIs sequentially, while eleven of them received their CIs simultaneously. The participants’ other clinical details are presented in Table 1.

**Table 1:** Demographic and clinical characteristics of the participants

	M ± SD	Median (IQR)	Minimum	Maximum
Chronological age	60.73 ± 9.74	–	48	81
Age of diagnosis	–	1 (3)	1	24
Age of 1st CI	–	18 (11)	9	30
Age of 2nd CI	27.54 ± 8.13	–	12	38
Age at special education	–	36 (12)	24	60

### Measurement outcomes

#### TELD-3 outcomes

The mean receptive language standard score was 85.53 ± 16.77 (min: 52, max: 112), and the mean expressive language standard score was 84.83 ± 21.69 (min: 50, max: 119). When the degree of spoken language development was examined, 30% (*n* = 9) of the participants were very poor, 10% (*n* = 3) were poor, 16.7% (*n* = 5) were below average, 33.3% (*n* = 10) were average, and 10% (*n* = 3) at the above average degree.

#### FAPCI outcomes

The assessment form has a maximum score of 115 and a minimum score of 23. The participants in this study had a mean FAPCI score of 93.77 ± 14.81, with a minimum score of 60 and a maximum score of 114.

#### SSES outcomes

The SSES has a maximum score of 310 and a minimum score of 62. The participants in this study had a mean SSES score of 198.67 ± 45.15, with a minimum score of 116 and a maximum score of 290.

#### BOT-2 outcomes

In the Manual Dexterity subtest, the mean standard score of the participants was found 11.37 ± 5.40. When the category of participants was examined, 10% of the participants were well-below average, 50% were below average, 30% were average, and 10% were above average category.

In the Bilateral Coordination subtest, the mean standard score of the participants was found 20.37 ± 4.59. When the category of participants was examined, 40% of the participants were average, 40% were above average, and 20% were well-above average category.

### Correlation analysis outcomes

**Fig. 1:**
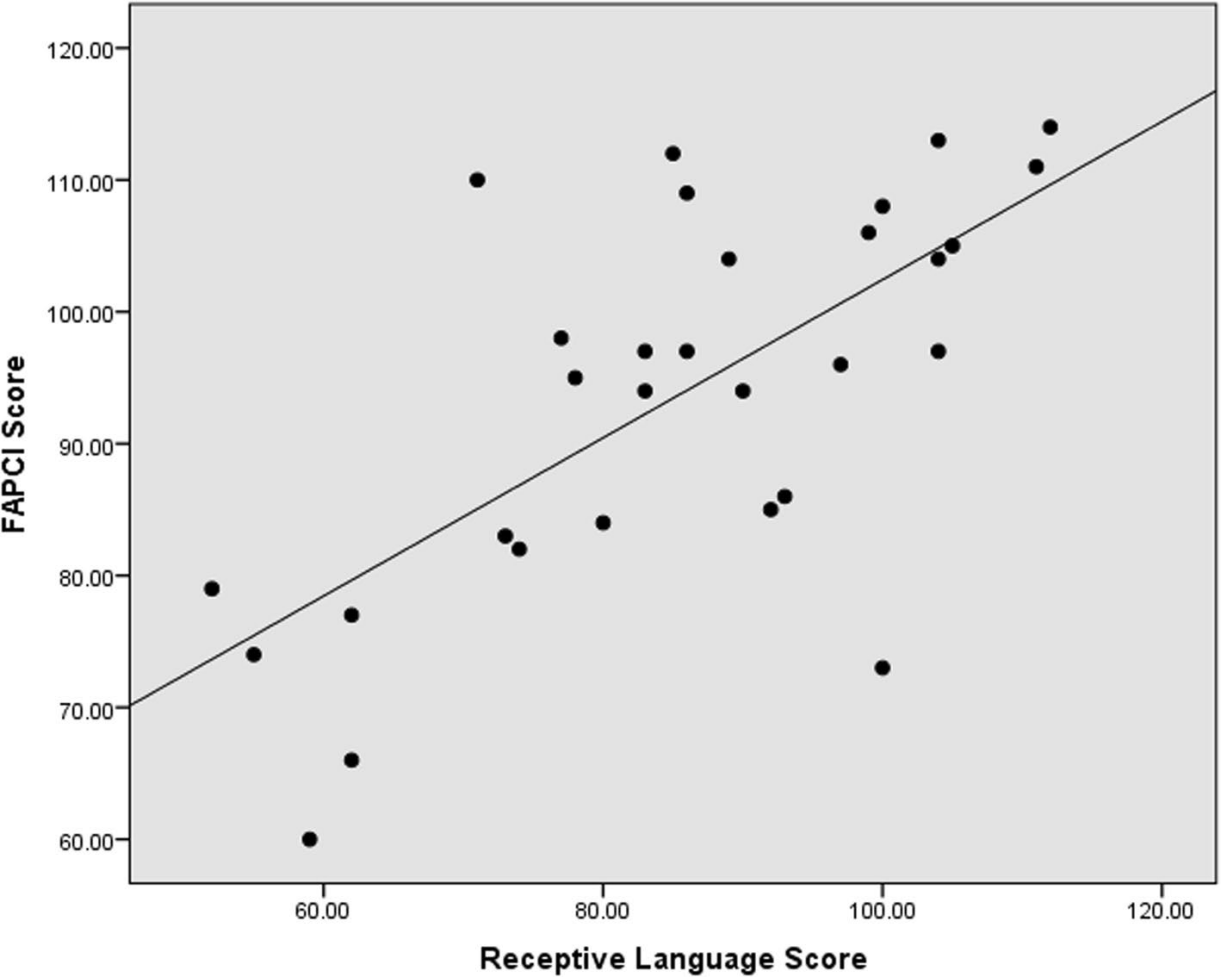
The correlation between FAPCI and Receptive Language scores. The analysis showed a significant positive correlation with a correlation coefficient of *r* = 0.67 and *p *value = 0.00

**Fig. 2:**
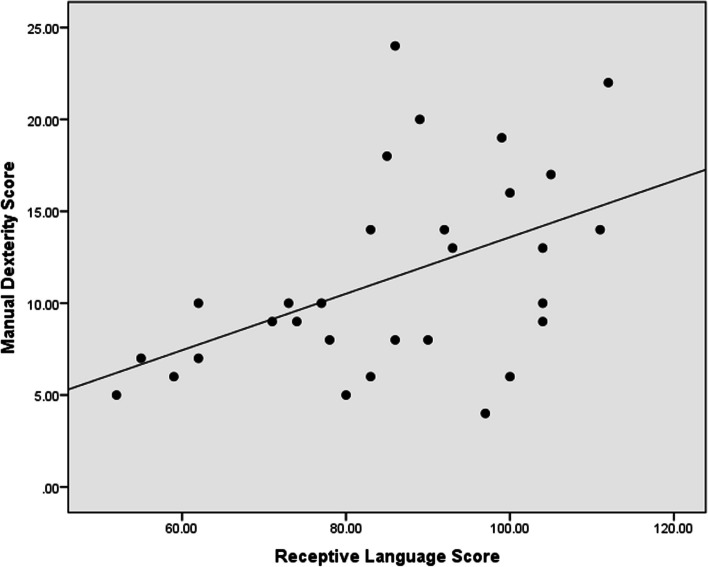
The correlation between Manual Dexterity and Receptive Language scores. The analysis showed a significant positive correlation with a correlation coefficient of *r* = 0.47 and *p *value = 0.008

**Fig. 3:**
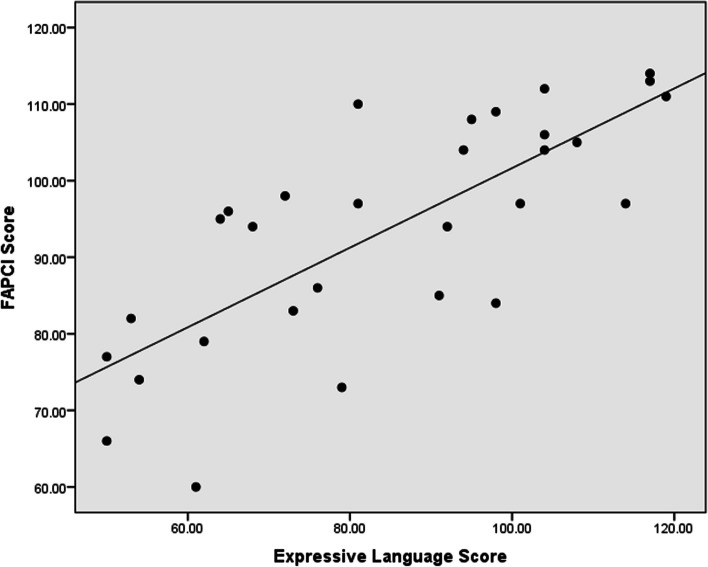
The correlation between FAPCI and Expressive Language scores. The analysis showed a significant positive correlation with a correlation coefficient of *r* = 0.76 and *p *value = 0.00

**Fig. 4:**
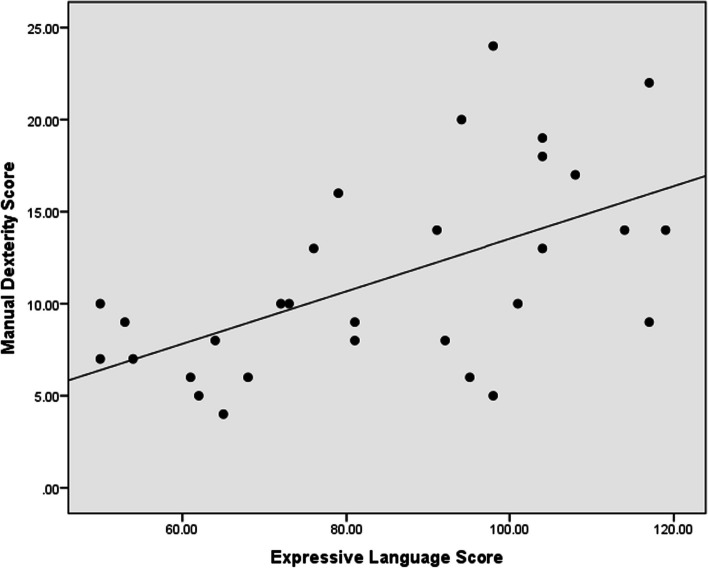
The correlation between Manual Dexterity and Expressive Language scores. The analysis showed a significant positive correlation with a correlation coefficient of *r* = 0.57 and *p *value = 0.001

**Fig. 5:**
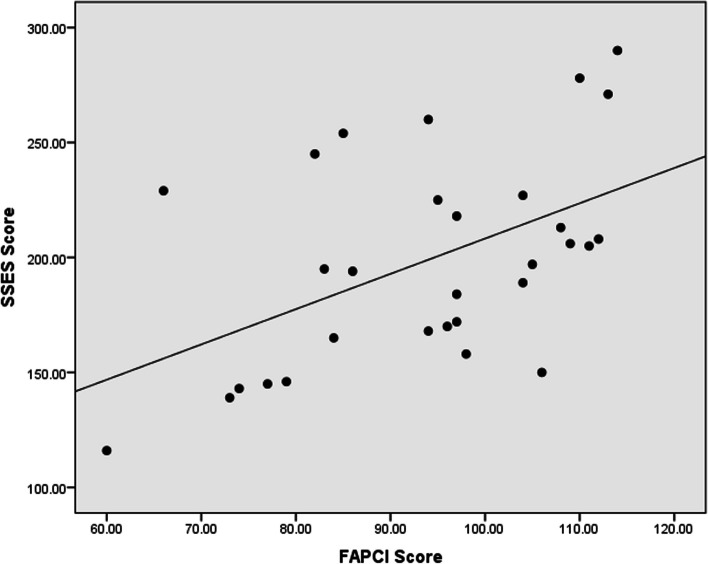
The correlation between SSES and FAPCI scores. The analysis showed a significant positive correlation with a correlation coefficient of *r* = 0.70 and *p *value = 0.005

**Fig. 6:**
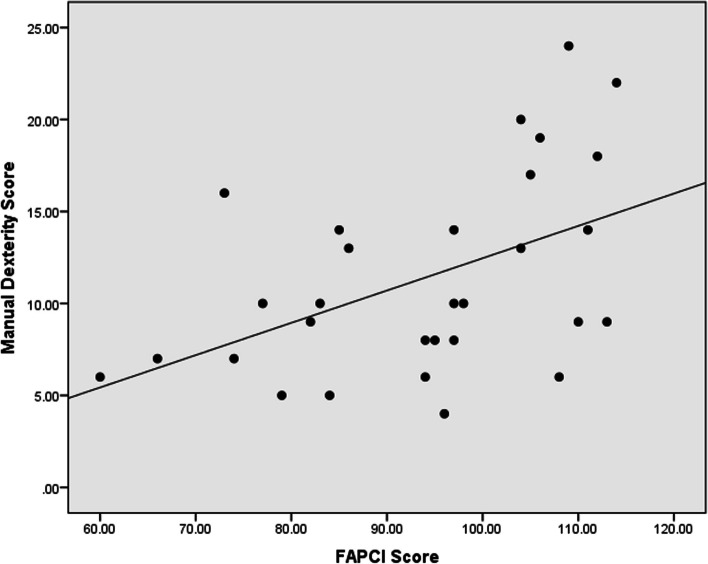
The correlation between Manual Dexterity and FAPCI scores. The analysis showed a significant positive correlation with a correlation coefficient of *r* = 0.03 and *p *value = 0.006

In the correlation analysis, the receptive language has a strong positive correlation with FAPCI scores (*r* = 0.67, *p* < 0.001) and a moderate positive correlation with Manual Dexterity (*r* = 0.47, *p* = 0.008) (Figs. 1, 2). Similarly, expressive language has a strong positive correlation with FAPCI scores (*r* = 0.76, *p* < 0.001) and a moderate positive correlation with the Manual Dexterity (*r* = 0.57, *p* = 0.001) (Figs. 3, 4). The FAPCI has a strong positive correlation with SSES (*r* = 0.70, *p* = 0.005) and a weak positive correlation with Manual Dexterity subtest scores (*r* = 0.03, *p* = 0.006) (Figs. 5, 6). There was no significant correlation found between the Bilateral Coordination subtest scores of the participants and their scores in language, FAPCI, and SSES (Table 2).

**Table 2:** Correlation analysis outcomes

Variables	Receptive language		Expressive language		FAPCI		SSES		Manual dexterity		Bilateral coordination	
	*p*	*r*	*p*	*r*	*p*	*r*	*p*	*r*	*p*	*r*	*p*	*r*
Receptive language	–	–	–	–	0.00***	**0.67**	0.07	0.32	0.008**	**0.47**	0.28	0.20
Expressive language	–	–	–	–	0.00***	**0.76**	0.13	0.28	0.001**	**0.57**	0.64	0.08
FAPCI	0.00***	**0.67**	0.00***	**0.76**	–	–	0.005***	**0.70**	0.006*	**0.03**	0.81	0.27
SSES	0.07	0.32	0.13	0.28	0.005***	**0.70**	–	–	0.50	0.12	0.68	0.07
Manual dexterity	0.008**	**0.47**	0.001**	**0.57**	0.006*	**0.03**	0.50	0.12	–	–	–	–
Bilateral coordination	0.28	0.20	0.64	0.08	0.81	0.27	0.68	0.07	–	–	–	–

Bold indicates significant correlation, * positive week correlation, ** positive moderate correlation, *** positive strong correlation

## Discussion

This study investigated the relations between language abilities, functional hearing, social skills, bilateral coordination and manual dexterity skills in children with early CIs. Because the brain is an interconnected operating system, it is possible that an early period of auditory deprivation may have secondary neurological effects in addition to the obvious hearing-related effects [14]. Therefore, it is important to investigate the non-auditory and non-verbal abilities along with auditory and language skills in children with CIs.

CIs provide a significant advantage for individuals with prelingual HL through supporting the speech and language development [23]. In this study, we found that the degree of spoken language development of participants were mainly average. This is encouraging evidence in regards to the potential benefits of implantation. Kutlu et al. [24] noted that the acquisition of receptive and expressive language skills and functional hearing are related to each other. Functional hearing is about the how individual use their hearing to gather information, how they make sense of this information in various environments throughout the day [25]. Hence, evaluating the functional hearing enables us to gather insights into the communication skills of children in their daily lives. The study revealed the expected finding of a significant correlation between the receptive and expressive language and the functional hearing. This suggests that children with well-developed functional hearing skills are able to acquire language skills more quickly, enabling them to use their receptive and expressive language more efficiently.

The body of research indicates that the process of acquiring language is highly related to perceptual, computational, social, and neural systems, which also involve the motor development [26, 27]. It was suggested that early motor development provide opportunities for language acquisition supporting interaction and social engagement [28]. At the same time, the early exposure to language and the development of motor skills have a significant impact on the development of language acquisition, emphasizing important associations between objects, actions, and thoughts [27]. Brooks et al. [29] explained this connection via a neuropsychological approach in which brain regions associated with motor control, speech, and language abilities are connected. Several studies have shown disturbances in various aspects of motor performance in children with CIs. For example, Schlumberger et al. [30] and Conway et al. [14] found in their study that children with CIs experienced difficulties in the development of complex motor sequence production. In this study, we evaluated the participants’ manual dexterity skills using the Manual Dexterity subtest, which requires fine motor skills. The findings indicated that the participants’ manual dexterity development was generally below the typical norm values. The study also showed an association between manual dexterity skills and receptive and expressive language skills, as well as functional hearing. Similarly to the current study, Aslan et. al. [31] used to TELD-3 and BOT-2 to measure the language skills and motor development of children with inner ear malformations who received CIs. They found a significant correlation between participants’ motor skills and language development. In their review study, Gonzalez et al. [27] stated that most of the studies in the literature have established a significant relation between fine motor skills and language abilities. Additionally, they claimed that language studies focus more on gross motor skills than fine motor skills [27]. The relation between bilateral coordination skills, which mostly encompass gross motor skills, and language and functional hearing abilities was also investigated in this study, and no significant relationship was found. The findings demonstrated that participants’ bilateral coordination abilities were generally average or above average when compared to typical norm values. According to existing literature, gross motor abilities have a significant relationship with language abilities [32–34]. However, the majority of studies have been conducted during infancy and toddlerhood rather than childhood, which limits our interpretation of findings for older age ranges. In addition, Conway et al. [14] suggested that auditory deprivation can disrupt aspects of non-verbal cognition, particularly skills related to visual-motor patterns, even if hearing is restored with a CI. Because, visual-motor abilities require representation and organization of serial task [14]. As a result, many children with CIs are still likely to experience delays in the development of neural pathways that underlying these information processing systems [14, 35]. In this study, the Manual Dexterity subtest includes tasks that require higher visual-motor abilities than the Bilateral Coordination subtest. It was thought that this discrepancy in task requirements could explain the participants' more difficulty in the Manual Dexterity subtest in comparison to the Bilateral Coordination subtest.

Further, we evaluated the participants’ social skills in this study. Because social skills are crucial for the development of joint attention, reciprocation, and awareness of others' intentions and hearing-impaired children have social difficulties when compated to their NH peers [36]. Research has shown that CIs enable children with hearing impairments to prevent social isolation and actively engage in social interactions [37, 38]. The widely recognized advantage of the CIs mainly results from their impact on language and functional auditory abilities [16, 39]. The study revealed a significant correlation between the functional hearing abilities and social skills. However, there was not a significant relationship between social skills and receptive and expressive language abilities. Given the existing studies and the close connection between social and language skills [16, 38, 40], we were expecting to find a significant relationship between receptive and expressive language skills and social skills. Considering the children attended various special education services, schools, and kindergartens, SSES was rated by different teachers. We predicted that differences among raters could have subjectively affected the outcomes. Furthermore, it was observed that a variety of evaluation tools are used in research performed to evaluate social skills. This could be considered a contributing factor to the differences between the obtained results and the results reported in the current body of literature.

The study also examined the correlation between social skills and motor developmental skills, but no significant relationship was found. Studies have indicated a correlation between the development of the motor system and social interaction in children with typically developing and neurodevelopmental disorders [41, 42]. Especially, it was stated that gross motor skills predict social function in children with autism spectrum disorders [41]. In children with CIs, no previous research has investigated the correlation between the motor development abilities and social skills in the literature. Therefore, further research is needed to investigate the relationship between social abilities and motor development skills in children with CIs.

There were several limitations in this study. We did not include a control group with normal hearing. It was the main limitation of our study. Including normal-hearing group would have strengthened this study as it allowed comparisons between two groups. Furthermore, the study might have been strengthened by including children with severe to profound congenital hearing loss who did not use any amplification devices or only used hearing aids. This would have allowed for a more comprehensive evaluation of the early cochlear implant’s impact on language development, functional hearing abilities, social abilities, and motor development skills. Second, the participants were not homogenous in terms of amplification use. Six of the participants were unilateral CI users, and 24 were bilateral users. Additionally, to reflect the development of children with CIs, these assessments and analyses should be performed before and after implantation.

In sum, the present study provide an opportunity to examine the relations between functional hearing, language, social, bilateral coordination and manual dexterity development skills in children with early CIs. Our findings suggest that although children with CIs show age-typical levels of performance on bilateral coordination skills, they appear to be impaired on manual dexterity skills. Furthermore, manual dexterity skills, functional hearing abilities and receptive and expressive language outcomes were closely related with each other in these children. These findings suggest that professionals need to use a holistic approach in the evaluation and rehabilitation of children with CIs.

## Data Availability

Not applicable.
